# Model for Prioritizing Best Management Practice Implementation: Sediment Load Reduction

**DOI:** 10.1007/s00267-012-9977-4

**Published:** 2012-11-11

**Authors:** Taeil Jang, George Vellidis, Jeffrey B. Hyman, Erin Brooks, Lyubov A. Kurkalova, Jan Boll, Jaepil Cho

**Affiliations:** 1 Department of Rural Construction Engineering, Chonbuk National University, Jeonju-si, Jeonbuk 561-756 Republic of Korea; 2 Crop & Soil Sciences Department, University of Georgia, 2360 Rainwater Road, Tifton, GA 31793-5766 USA; 3 Conservation Law Center, Bloomington, IN 47408 USA; 4 Biological and Agricultural Engineering Department, University of Idaho, Moscow, ID 83844 USA; 5 Department of Economics and Energy and Environmental Systems Program, North Carolina A&T State University, Greensboro, NC 27411 USA; 6 Biological and Agricultural Engineering Department, University of Idaho, Moscow, ID 83844 USA; 7 Climate Change Research Team, Climate Research Department, APEC Climate Center, Haeundae-gu, Busan, 612-020 Republic of Korea

**Keywords:** Synoptic assessment, Geographic prioritization, Conceptual model, Sediment, Conservation practices, Indicator, CEAP

## Abstract

Understanding the best way to allocate limited resources is a constant challenge for water quality improvement efforts. The synoptic approach is a tool for geographic prioritization of these efforts. It uses a benefit-cost framework to calculate indices for functional criteria in subunits (watersheds, counties) of a region and then rank the subunits. The synoptic approach was specifically designed to incorporate best professional judgment in cases where information and resources are limited. To date, the synoptic approach has been applied primarily to local or regional wetland restoration prioritization projects. The goal of this work was to develop a synoptic model for prioritizing watersheds within which suites of agricultural best management practices (BMPs) can be implemented to reduce sediment load at the watershed outlets. The model ranks candidate watersheds within an ecoregion or river basin so that BMP implementation within the highest ranked watersheds will result in the most sediment load reduction per conservation dollar invested. The model can be applied anywhere and at many scales provided that the selected suite of BMPs is appropriate for the evaluation area’s biophysical and climatic conditions. The model was specifically developed as a tool for prioritizing BMP implementation efforts in ecoregions containing watersheds associated with the USDA-NRCS conservation effects assessment project (CEAP). This paper presents the testing of the model in the little river experimental watershed (LREW) which is located near Tifton, Georgia, USA and is the CEAP watershed representing the southeastern coastal plain. The application of the model to the LREW demonstrated that the model represents the physical drivers of erosion and sediment loading well. The application also showed that the model is quite responsive to social and economic drivers and is, therefore, best applied at a scale large enough to ensure differences in social and economic drivers across the candidate watersheds. The prioritization model will be used for planning purposes. Its results are visualized as maps which enable resource managers to identify watersheds within which BMP implementation would result in the most water quality improvement per conservation dollar invested.

## Introduction

Sediment is ranked as the number one pollutant of surface waters in the United States (EPA 1996). Excessive sediment in surface water causes problems for aquatic life by increasing turbidity and destroying habitat; increases treatment costs for drinking water plants, industrial users, and some agricultural users; and reduces recreational opportunities (EPA 1996; Vellidis and others 2003b). The methods for reducing sediment loading to streams in agricultural landscapes have been studied extensively (Lowrance and others 1984; Babcock and others 1996; Vellidis and others 2003a; McKergow and others 2003; Borah and others 2006; Matthew and others 2009; Kling 2011). As a result, many conservation practices have been developed over the past 50 years to reduce erosion and the US Department of Agriculture (USDA)-Natural Resources Conservation Service (NRCS) has been at the forefront of these efforts. Over the past five decades, NRCS has provided hundreds of billions of dollars in cost-share assistance for conservation programs (Monke and Johnson 2010; ACMWG 2011). For example, since 1987, the NRCS conservation reserve program alone has distributed $29.7 billion to owners of agricultural land to implement conservation practices that reduce soil loss, restore wetlands, and conserve forested areas (USDA 2006).

In order to improve surface water quality, NRCS typically identifies watersheds with water quality problems and develops cost-share programs to encourage land operators within the watershed to adopt conservation practices. The watersheds may range in size from a few hundred km^2^ to the Mississippi River Basin. The watersheds are selected primarily by the magnitude of their observed water quality problems. Within the watershed, cost-share resources are available to all landowners rather than being focused on priority areas within the watershed.

Under a geographic prioritization scheme, resources are allocated to watersheds and within watersheds where the functional benefits from implementation are the greatest (Babcock and others 1996; Hyman and Leibowitz 2000; McAllister and others 2000; Vellidis and others 2003a; Feng and others 2006). In other words, geographic prioritization attempts to allocate resources to the areas where best management practices (BMP) implementation results in the most water quality improvement for a given conservation budget. The geographic prioritization scheme can be applied to many scales ranging from areas within a relatively small watershed to watersheds within an ecoregion or river basin.

The early economic literature on cost-effective BMP placement relied on relatively simple models of water quality that assumed that the effectiveness of BMPs can be assessed on a field-by-field (or subwatershed-by-subwatershed) basis (Babcock and others 1996). More recent analyses began incorporating more realistic hydrological modeling into the spatial optimization framework (Shortle and Horan 2001; Khanna and others 2003; Kling 2011). Optimization of BMP placement within watersheds using complex hydrological models and heuristic algorithms has recently been demonstrated by many researchers (Bekele and Nicklow 2005; Arabi and others 2006; Maringanti and others 2009; Pandey and others 2009; Rodriguez and others 2011). The models require large detailed datasets for the parameterization and validation of their hydrologic and economic components. These detailed datasets are not available for most watersheds and developing them requires significant amounts of time and resources.

Because of these limitations, several simpler and less resource-intensive prioritization concepts and procedures have been developed. Hruby and others (1995) describe the indicator value assessment, a rapid assessment procedure that considers wetland values on a regional scale. Llewellyn and others (1996) studied a restoration planning procedure for prioritizing existing wetland forest patches, and Walter and others (2000) suggested the term of HSA (hydrological sensitive area) for identifying water quality risk reduction targets. Machado and others (2006) presented a framework to prioritize conservation investments by considering social benefits, with the objective of supporting farmland preservation programs. Feng and others (2006) studied the optimal placement of more than one BMP under a single conservation budget. Khare and others (2007) evaluated a logical approach for prioritizing watersheds on the basis of a soil erosion status index. Norton and others (2009) developed a restorability screening approach using recovery-relevant ecological, stressor, and social context metrics for prioritizing restoration efforts.

The synoptic approach, first proposed by Leibowitz and others (1992), was originally developed for the geographic prioritization of ecological restoration efforts. Synoptic refers to general view of a whole, and a synoptic approach, therefore, provides a broad perspective rather than a detailed analysis (Abbruzzese and Leibowitz 1997). A synoptic approach provides a compromise between the need for rigorous results and the need for timely information, and is specifically designed to incorporate the best professional judgment in cases where information and resources are limited. The synoptic approach uses a benefit-cost framework to calculate indices for functional criteria in subunits (watersheds, counties, etc.) of a region and then to rank the subunits (Hyman and Leibowitz 2000). To date, the synoptic approach has been applied primarily to local or regional wetland restoration prioritization projects by Abbruzzese and Leibowitz (1997), Hyman and Leibowitz (2000), McAllister and others (2000), and Vellidis and others (2003a).

Our objective was to develop a model which uses the synoptic approach for prioritizing watersheds within which agricultural BMPs can be implemented to reduce sediment load at the watershed outlets. The model was specifically developed as a tool for prioritizing BMP implementation efforts in the ecoregions containing the 17 watersheds associated with the USDA-NRCS conservation effects assessment project (CEAP). Thus unlike other applications of the synoptic approach, this model can be applied nationally under a wide variety of biophysical and climatic conditions. Our long-term goal is for this model to be adopted by agencies such as NRCS and used for planning and resource allocation decisions.

## Methods

### Prioritization Criterion

**Fig. 1:**
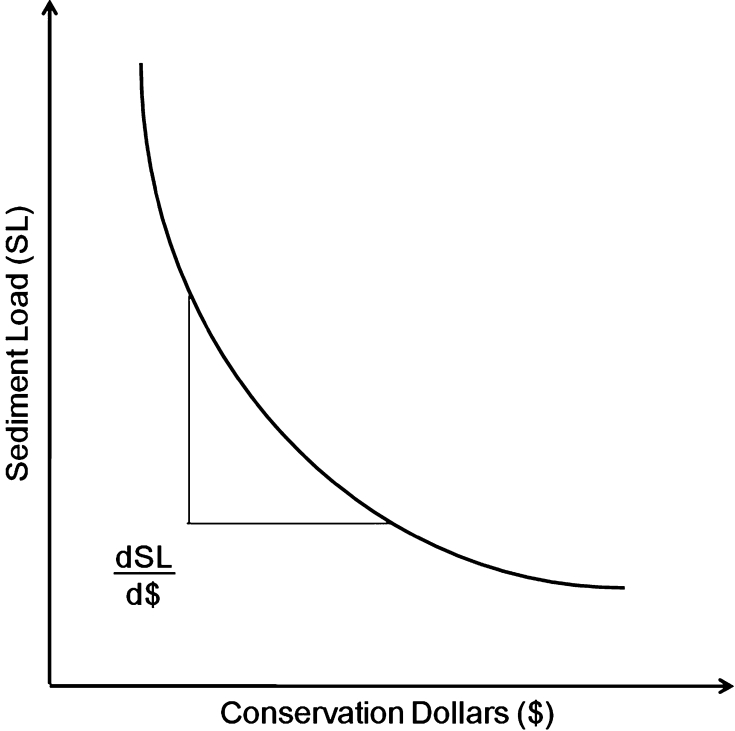
Hypothesized relationship between sediment load and invested conservation dollars (Vellidis and others 2003a)

A synoptic approach utilizes a prioritization criterion to comparatively rank BMP implementation options. This prioritization criterion is generally expressed as the ratio of the marginal change in ecological function per conservation dollar invested. For the sediment load reduction case, the prioritization criterion becomes the marginal change in total sediment load, dSL (kg/km^2^/year), per conservation dollar invested (d$), or dSL/d$. We anticipate this ratio to be negative—that is, we expect a marginal decrease in sediment load per conservation dollar invested. We also anticipate a nonlinear, convex relationship between the absolute value of dSL/d$ and the total conservation investments as illustrated in Fig. 1 reflecting the commonly observed decreasing marginal benefit schedule (Tietenberg 2006). When only a single suite of BMPs is considered, rank-ordering subwatersheds by dSL/d$ from the highest to the lowest in absolute value and then selectively placing the BMPs in the subwatersheds from the top of the list until a conservation budget is exhausted results in the maximum pollution reduction for the given conservation budget (Babcock and others 1996). The criterion results in the cost-effective use of the conservation budget only if the total effort is constrained and various implementation efforts offer functional equivalence (Hyman and Leibowitz 2000). In other words, if terracing field A or field B results in equivalent sediment yield reduction, and if we choose to terrace field B because it is more cost-effective, we still achieve the desired sediment yield reduction.

Change in total sediment load is not only a function of the area conserved but also a function of the hydrologic responses of the watershed. The hydrologic response is characterized by hydrologic processes involved precipitation, surface runoff, infiltration/percolation, sediment detachment-transport-deposition, etc. Improved hydrologic response is also defined as a “decrease” or “attenuation” in hydrologic response. Increased marginal attenuation of the hydrologic response of a watershed is primarily a function of the marginal change in conserved area of a watershed. This process can be expressed mathematically by applying the chain rule:

1 $$ \frac{{{\text{dSL}}_{\text{j}} }}{{{\text{d\$ }}_{\text{j}} }} = \frac{{{\text{dCA}}_{\text{j}} }}{{{\text{d\$ }}_{\text{j}} }} \times \frac{{{\text{dSL}}_{\text{j}} }}{{{\text{dCA}}_{\text{j}} }} $$

where,

$\frac{{{\text{dSL}}_{\text{j}} }}{{{\text{d\$ }}_{\text{j}} }}$ is the marginal change in total sediment load per conservation dollar invested in subwatershed j,

$\frac{{{\text{dCA}}_{\text{j}} }}{{{\text{d\$ }}_{\text{j}} }}$ is the marginal change in conserved area per conservation dollar invested in subwatershed j, and

$\frac{{{\text{dSL}}_{\text{j}} }}{{{\text{dCA}}_{\text{j}} }}$ is the marginal change in sediment load per conserved area *j*.

### Descriptors and Indicators

Equation 1 depicts the mathematical formulation of the conceptual model that links our ecological endpoint (sediment load reduction per conservation dollar invested) with the descriptors selected to prioritize watersheds. Each term of Eq. 1 is defined by a number of descriptors which can be assessed using a set of indicators with described measurement endpoints and available data sources.

Indicators are useful when the ecological endpoint is difficult or costly to measure directly, the decision risk is low, and the management concern calls for a relative rather than complete assessment of alternatives (Abbruzzese and Leibowitz 1997; Schweiger and others 2002). Selecting indicators through a conceptual model, rather than based on data availability, helps avoid the use of information that is not relevant (McAllister and others 2000) and also helps identify redundant indicators as well as important descriptors for which data are not available. We followed the judgement-based structural equation modeling (JSEM) approach developed by Hyman and Leibowitz (2001) for selecting and evaluating indicators. JSEM is a quantitative framework for structuring and evaluating information about relationships between indicators and an endpoint, where this information may be based on expert judgment, to identify and evaluate potential indicators Hyman and Leibowitz (2001).

Our descriptors and indicators represent the social, economic, and hydrologic drivers of sediment load reduction within a watershed and are correlated with those reported by the literature and through consultation with appropriate professionals (Lowrance and Vellidis 1995; Abbruzzese and Leibowitz 1997; Walter and others 2000; Hyman and Leibowitz 2001; Vellidis and others 2003a; Machado and others 2006; Khare and others 2007).

1. Marginal change in conserved area per conservation dollar Invested, dCA/d$

Our model assumes that a positive marginal change, or increase, in conserved area will result from the expenditure of conservation dollars invested. The term dCA/d$ is used to assemble the descriptors that are important for assessing the increase in conserved area that can be achieved per conservation dollar. This term is a function of the community’s support and willingness to engage in conservation activities and the efficiency of BMP implementation within a watershed and can be expressed as follows:

2 $$ \begin{gathered} \frac{{{\text{dCA}}_{j} }}{{{\text{d\$ }}_{\text{j}} }}\, = f ( {\text{community support and willingness for conservation activities}}, \, \hfill \\ {\text{ BMP implementation factors)}} \hfill \\ \end{gathered} $$

Support and willingness for conservation activities and BMP implementation factors are the two descriptors for this term. The descriptors and their indicators, measurement endpoints, and potential data sources are given in Table 1 and discussed in more detail below. Only measurement endpoints that adequately represent the indicators and for which data sources are readily available were selected.

**Table 1:** Descriptors, indicators, measurement end points, and data sources for the marginal change in conserved area per conservation dollar invested (dCA/d$)

Descriptors	Indicators	Measurement endpoints	Data sources
Community support and willingness for conservation activities	Watershed protection activities	Density of watershed protection groups	USDA-NRCS, EPA, local govt.
		Density of environmental group chapters	National, state offices of environmental groups, web sites
	Conservation programs	Areas protected by conservation easements or similar activities	USDA-NRCS, state environmental regulatory agency, local govt.
BMP implementation factors	Implementation cost	Cost of conservation actions	County tax offices, US Census of Agriculture
	Land availability	Conservation practice areas stability and disturbance	USDA-NRCS land use maps

#### Community Support and Willingness for Conservation Activities

This descriptor is a qualitative measure of the watershed residents’ disposition toward watershed conservation activities and was described by Norton and others (2009) as the social context affecting efforts to improve a watershed’s condition. In general, water quality improvement projects are more likely to succeed in watersheds with high support and willingness for conservation activities (Norton and others 2009). The US Environmental Protection Agency (EPA) has developed a list of social context indicators (USEPA 2011) from which we selected those indicators most relevant to our model—the density of active watershed protection groups and environmental group chapters. For example, grassroots watershed protection (Adopt-A-Stream, Adopt-a-Watershed), environmental groups (Sierra Club, Audubon Society), or watershed councils are all indicators of community support and willingness for conservation activities because residents of watersheds with these types of activities may be more willing to participate in conservation easements or sell land designated for conservation activities below market value and the conservation costs may be reduced by volunteer activities from environmental group members (USEPA 2011).

Another indicator of this descriptor is the presence of land conservation programs such as easement programs on private land (e.g., federal easements, land trust easements). These activities are an indicator of the prospects for a given proportion of total watershed land area to remain in conditions desirable for water quality restoration and protection (USEPA 2011).

#### BMP Implementation Factors

Best management practices implementation costs and land availability are the two principal indicators of this descriptor. Conceptually, the cost of implementing conservation practices on agricultural lands is the minimum monetary payment that a farmer is willing to accept to install and maintain the practice in question. This opportunity cost includes the direct explicit cost of physically installing and managing a conservation practice and may additionally include the revenue lost by diverting the land from agricultural production to a conservation use, the cost of learning about the practice, and the costs associated with the uncertainty surrounding the decision. Complex socio-economic drivers such as farm size, farmer’s age and/or gender, renting status, and other farm- and farmer-specific characteristics may affect the location- and farmer-specific costs of conservation practice selection, adoption, and effectiveness (Pannell and others 2006; Prokopy and others 2008).

Natural Resources Conservation Service (NRCS)-driven conservation practices have already been installed on many agricultural lands. Therefore, land available for additional conservation practices is important in ranking watersheds—when there are few existing NRCS programs in a watershed, there is more land available for new conservation actions.

2) Marginal change in sediment load per conserved area, dSL/CA

As conserved area is increased within a watershed, a corresponding decrease (improvement) in hydrologic response can be expected. Improved hydrologic response results in reduced flow velocities and, consequently, reduced sediment load. This term, dSL/dCA, is used to assemble the descriptors that are important for assessing the marginal decrease in sediment load that can be achieved as conserved area on agricultural lands is increased. In this study we consider only sediment derived from agricultural lands. Sediment load from agricultural lands is a function of many factors including land cover, agricultural production methods, soil type, slope, and precipitation patterns. Indicators and data for measurement endpoints that integrate these functions are not readily available. Simple erosion prediction models, however, do integrate these functions, and we use such a model to estimate dSL/dCA. These models are relatively easy to apply and, therefore, useful for calculating watershed sediment loads, which can then be used for quantitative ranking (Walter and others 2000; Vellidis and others 2003a).

#### Hydrologic Characterization Tool (HCT)

In this study, hydrologic and sediment response within a watershed was estimated using the HCT (Brooks and others 2010; Brooks and Boll 2011). The HCT is a web-interface program which uses a modified version of the water erosion prediction project (WEPP, Laflen and others 1991) model (Boll and others 2011) to identify the effects of various management practices on hydrologic flow paths and sediment transport through specific land types in a region. The model simulates runoff, subsurface lateral flow, percolation, soil detachment, transport, and delivery of sediment by overland flow by representing hillslopes as three linear segments—the upper, middle, and lower parts of the slope. A land type is defined by a unique combination of soil, climate, and topographic attributes based on user selections. Users also select the crop rotation, type of tillage operation (i.e., conventional, conservation, or no-till), and potential management practices (i.e., grass buffer strips) that could be potentially applied to each land type in each region. Using this information, the HCT provides average annual and monthly output for each land type for all possible management practices in the region. Output from the model can then be linked back to the predefined land types using geographical information system (GIS) to map critical management zones within a watershed. Like the interface tools developed by Elliot (2004), the HCT was not developed to simulate complex hillslopes. However, limiting the flexibility to a few essential parameters simplifies the model and makes the tool easier to learn and apply over a wide range of conditions. Brooks and others (2011) provide a detailed description of the HCT.

### Model Implementation

**Fig. 2:**
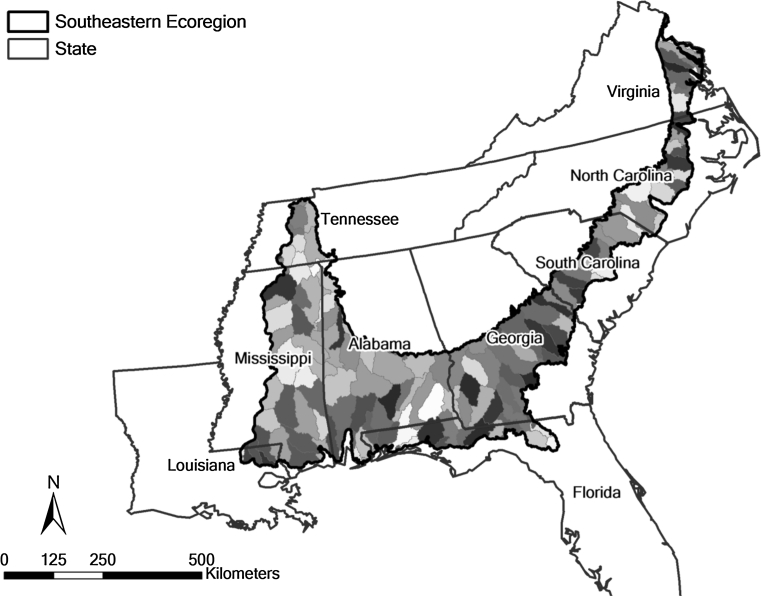
The ecoregion of the southeastern coastal plain with superimposed boundaries of the 8-digit hydrologic unit code (HUC) watersheds

The first step in implementing the prioritization model is setting the geographic boundaries. Within this geographical boundary, the resource specialist performing the prioritization must decide on the appropriate scale. As an example, we assume that the scale will be 8-digit hydrologic unit code (HUC) watersheds in the ecoregion of the southeastern coastal plain shown in Fig. 2. This is one of the ecoregions to which the model will be applied and which contains the little river experimental watershed (LREW), the site of the Georgia CEAP project. HUC watersheds are delineated by the U.S. Geological Survey using a nationwide system based on surface hydrologic features. The goal is to prioritize the 161 HUC-8 watersheds in this ecoregion so that available conservation dollars will be invested strategically while maximizing sediment load reduction.

The next step is to develop the mathematical expressions that will combine the descriptors that define each term of Eq. 1. The equation’s descriptors and their associated indicators were described in the previous paragraphs. For the coastal plain example, the equation must be quantified for all 161 8-digit HUCs in the ecoregion. We used the standard combination rules for individual descriptors and indicators as discussed by Skutch and Flowerdew (1976), Hopkins (1977), O’Banion (1980), FWS (1981), Smith and Theberge (1987), Abbruzzese and Leibowitz (1997), Leibowitz and Hyman (1999), and Hyman and Leibowitz (2001).

The subsequent paragraphs describe how we developed the mathematical expressions to combine descriptors and their indicators that define each term of Eq. 1.

1. Marginal change in conserved area per conservation dollar invested, dCA/d$

The marginal change in conserved area per conservation dollar invested is defined by two descriptors and is presented as:

3 $$ \left( {{\text{dCA}}_{\text{j}} /{\text{d}}\$_{\text{j}} } \right)' \, = {\text{ CW}}_{\text{j}} \times {\text{ CL}}_{\text{j}} $$

where (dCA_j_/d$_j_)’ represents the marginal change in conserved area per conservation dollar invested in subwatershed j, the descriptor CW_j_ represents community support and willingness for conservation activities, and the descriptor CL_j_ represents BMP implementation factors for subwatershed j. Each of these descriptors is further defined by indicators and/or measurement endpoints (Table 1), as shown in Eqs. 4–6.

The descriptor CW_j_ is determined from the following measurement endpoints:

4 $$ {\text{CW}}_{\text{j}} = w_{ 1} \times {\text{WP}}_{\text{j}} /{\text{WP}}_{ \hbox{Max} } + w_{ 2} \times {\text{ENVG}}_{\text{j}} /{\text{ENVG}}_{ \hbox{Max} } + w_{ 3} \times {\text{PREA}}_{\text{j}} /{\text{PREA}}_{ \hbox{Max} } $$

where WP_j_ is the density of watershed protection groups in subwatershed j, WP_max_ is the maximum density of watershed protection groups overall subwatersheds, ENVG_j_ is the density of environmental group chapters in subwatershed j, ENVG_max_ is the maximum density of environmental group chapters overall subwatersheds, PREA_j_ is the proportion of areas protected by conservation easements or similar activities in subwatershed j, PREA_max_ is the maximum proportion of areas protected by conservation easements or similar activities overall watersheds, and *w*_i_ is a weighting factor assigned by the best professional judgment of the model’s users or experts consulted by the users. The sum of the weighting factors (*w*_*i*_) should be 1. The weighting factor should be used to discriminate the importance of the measurement endpoints during the application of the model should this information be available. In the absences of such information, the weighting factor should be distributed equally, i.e., *w*_*i*_, = 0.333.

Cost of implementing conservation practices on agricultural lands is a function of the cost of physically installing and managing the conservation practice and the cost of the incentive required to induce landowners to adopt the practice. Complex socio-economic drivers typically play a role in establishing the level of incentive but, in general, establishing conservation practices is more cost-effective in areas where the incentive needed to induce adoption is lower.

The descriptor CL_j_ which represents BMP implementation factors is a function of two indicators and is defined as:

5 $$ {\text{CL}}_{\text{j}} = { 1}/{\text{CP}}_{\text{j}} \times {\text{ LA}}_{\text{j}} $$

where, CP_j_ is an indicator of the cost of implementing conservation practices within subwatershed j and LA_j_ is an indicator of land available for conservation within subwatershed j. LA_j_ is further defined as:

6 $$ {\text{LA}}_{\text{j}} = \, \left[ {\left( { 1- {\text{NRCA}}_{\text{j}} } \right) \, / \, \left( { 1- {\text{NRCA}}_{ \hbox{Min} } } \right) \, + \, \left( { 1- {\text{AGUR}}_{\text{j}} } \right) \, / \, \left( { 1- {\text{AGUR}}_{ \hbox{Min} } } \right)} \right]/ 2 $$

where NRCA_j_ is the proportion of area conserved through NRCS programs to the total area in subwatershed j, NRCA_min_ is the minimum proportion of area conserved through NRCS programs overall watersheds to the total area of all subwatersheds, AGUR_j_ is the proportion of agricultural and urban land use to the total area in subwatershed j, and AGUR_min_ is the minimum proportion of agricultural and urban land use overall subwatersheds to the total area of all watersheds.

2) Marginal change in sediment load per conserved area, dSL/CA

As conserved area is increased within a watershed, a corresponding decrease (improvement) in hydrologic response can be expected. The marginal change in sediment load per conserved area is defined as:

7 $$ \left( {{\text{dSL}}_{\text{j}} /{\text{dCA}}_{\text{j}} } \right)' \, = {\text{ SLOAD}}_{\text{j}} /{\text{ SLOAD}}_{ \hbox{Max} } $$

where (dSL_j_/dCA_j_)’ represents the marginal change in total sediment load per change in hydrologic response in subwatershed j, SLOAD_j_ is the sediment load in subwatershed j, and SLOAD_max_ is the maximum sediment load overall the subwatersheds. SLOAD_j_ is defined as the sum of all hillslopes simulated by the HCT in subwatershed j.

## Results

### Testing the Model on an Example Watershed

**Fig. 3:**
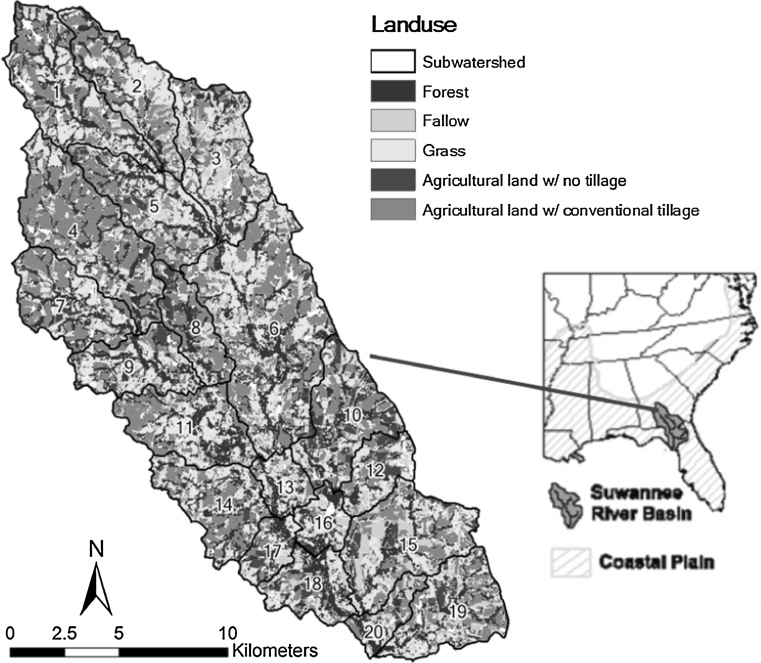
Land use map created from Landsat Thematic Mapper data with 30 × 30 m resolution showing the 20 subwatersheds in the Little River Experimental watershed (LREW)

As discussed earlier, our model will eventually be applied to all the ecoregions within which CEAP projects were conducted. It will be first applied to the southeastern Coastal Plain ecoregion containing the LREW (Fig. 2). We selected the LREW, located near Tifton, Georgia, USA, to test our prioritization model (Fig. 3) because in addition to being the site of complementary CEAP projects by the University of Georgia and by the USDA Agricultural Research Service (ARS) (Osmond 2010), it was selected by the ARS as a benchmark watershed representative of the southeastern coastal plain in the 1960s. Since 1968 it has been the subject of long-term hydrologic, water quality, and modeling research by USDA-ARS and the University of Georgia (Lowrance and others 1985; Lowrance and Smittle 1988; Sheridan 1997a, b; Bosch and others 2007a; Bosch and Sheridan 2007; Feyereisen and others 2007, 2008; Cho and others 2009, 2010a, b).

**Fig. 4:**
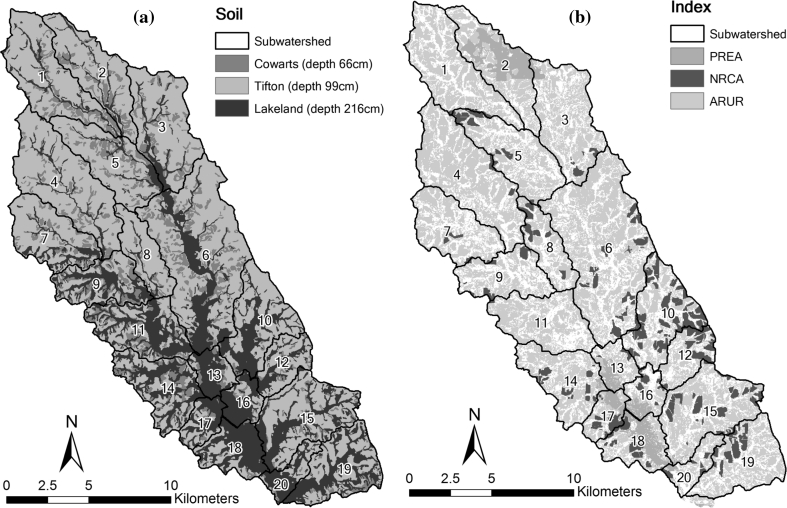
Maps of **a** depth to the hydrologically restrictive soil layer in the LREW using SSURGO data and **b** indices for areas protected by conservation easements (PREA), implemented conservation practices associated with NRCS programs (NRCA), and agricultural and urban areas (ARUR)

The climate of the LREW is humid subtropical with a long growing season (Bosch and others 2007b). Rainfall is unevenly distributed and often occurs as short-duration, high-intensity convective thunderstorms (Bosch and others 1999). The region has low topographic relief and is characterized by broad, flat alluvial floodplains, river terraces, and gently sloping uplands (Sheridan 1997a, b). Approximately 36 % of the land has less than 2 % slope, and only 7 % of the land has slopes in excess of 5 % (Cho and others 2010b). The soils are underlain by a plinthic layer of lower permeability at 0.9–1.5 m. Because of the plinthic layer which forces shallow lateral flow to the surface at lower elevations, the LREW landscape is dominated by a dense dendritic network of stream channels bordered by riparian forest wetlands (Sullivan and others 2007). The soils were defined as three main types based on the depth to the hydrologically restrictive soil layer using the NRCS soil survey geographic (SSURGO) database. The soil depths were identified as 0.66, 0.99, and 2.16 m (Fig. 4a).

Conservation activities have taken place in the LREW for several decades. Forty seven different BMPs have been implemented in the watershed with technical assistance by the NRCS and/or through federal cost-share conservation programs (Cho and others 2010b). An historical database of conservation practices adopted within LREW for 1970–2005 was created and entered into a GIS. The GIS database represents fields delineated according to farm tract using 1993 digital orthophoto quarter quadrangles. Each of the delineated fields contain information regarding program, conservation practice(s), implementation date, total acreage, expected lifetime of the practice, and cost-share versus non cost-share practices. These data may be queried to show spatial distributions by year, program, or practice. Figure 4b presents areas within LREW that are protected by conservation easements (PREA in Eq. 4) as well as areas on which conservation practices relevant to this project (discussed below) have been implemented via NRCS technical assistance and/or federal cost-share programs (NRCA in Eq. 6).

Forty-one percent of the LREW land area is in row crops and pasture as shown in Fig. 3 (Bosch and others 2006). From 1980 to 2003, conservation practices have been implemented on approximately 16 % of the land area in the LREW (Sullivan and Batten 2007). The areal extent of practices relevant to this project consisted of: grassed waterways (9.6 %), contour farming (9.5 %), conservation tillage (8.9 %), and terraces (8.8 %) (Sullivan and Batten 2007). Conservation practices may have also been applied to some of the remaining LREW cropped area by landowners without NRCS assistance. We do not have records of these practices; however, and for the purpose of this study we assume that no practices were implemented on this land area.

In order to test our model’s ability to prioritize watersheds, we divided the LREW into 20 sub-watersheds using GIS. The goal of the test application was to rank these subwatersheds. The watershed with the highest rank would provide the most sediment load reduction per conservation dollar invested.

### Marginal Change in Conserved Area per Conservation Dollar Invested, dCA/d$

We used the measurement endpoints and data sources listed in Table 1 for the descriptors contained in Eqs. 4–6. Watershed protection groups and environmental group chapters obtained from EPA watershed data (http://www.epa.gov/surf/) were used to quantify conservation support activities at the HUC-8 watershed scale or larger. However, since the LREW is relatively small and completely contained within a single HUC-8 watershed, the value assigned to WP_j_ and ENVG_j_ measurement endpoints was the same for all subwatersheds within LREW. Digital maps of areas protected by conservation easements were obtained from the Georgia GIS Clearinghouse (http://www.georgiaspatial.org). The local NRCS office provided information on actual BMP implementation costs within LREW. In this application we included the cost for terraces and grassed waterways, and two conservation practices that are important for controlling erosion in the LREW and for which it was relatively easy to vary costs based on terrain features. For slopes 2–5 %, the costs are $3,950 per 40 acres for terraces and $1,875 per acre for grassed waterways. For 5–7 % slopes the costs are $4,000 per 40 acres for terraces and $1,875 per acre for grassed waterways. Two other important practices—contour farming and conservation tillage—were not included because their implementation does not include fixed construction costs and is a function of farmer-dependent costs such as fuel and wear and tear of equipment.

The spatial distribution of implemented conservation practices associated with NRCS programs was obtained from datasets available from the USDA-ARS and University of Georgia (Sullivan and Batten 2007). Land available for conservation was determined using land use data obtained from the Georgia GIS clearinghouse and the spatial distribution of implemented conservation practices as described by Eq. 6. These data are displayed in Figs. 3 and 4. The CW_j_ and CL_j_ descriptors for each subwatershed were then calculated using Eqs. 3–6, and the results were used to rank the subwatersheds.

### Marginal Change in Sediment Load per Conserved Area, dSL/dCA

Table 2 presents annual erosion rates estimated with the HCT for the combination of hillslopes, depth to the hydrologically restrictive soil layer, and land use utilized for applying the model to the LREW. The erosion estimates are averages for a 30-year simulation period with generated climate conditions. The crop production areas were classified as fallow, conventional-tillage areas, or no-till. Predicted erosion rates were consistently greater for the shallowest depth to the hydrologically restrictive soil layer (0.66 m), conventional-tillage areas, and fallow areas. Erosion rates also increased consistently with slope.

**Table 2:** Comparison of annual erosion rate estimated with the Hydrologic Characterization Tool (HCT) for a 30-year simulation period with generated climate conditions

Slope^a^	Soil^b^	Erosion (kg/ha)				
		Forest	Fallow	Grass	Agric_NT^c^	Agric_CT^d^
Flat (2 %)	Shallow (66 cm)	118.3	70,213.5	779.1	7,105.9	30,243.7
Flat (2 %)	Mid (99 cm)	0.4	12,179.4	27.0	252.4	14,805.0
Flat (2 %)	Deep (216 cm)	0.0	2,802.1	0.0	0.0	9,099.3
Mod_Flat (5 %)	Shallow (66 cm)	123.3	94,513.2	813.5	10,452.8	4,2731.7
Mod_Flat (5 %)	Mid (99 cm)	5.4	11,648.7	53.8	445.3	1,6849.4
Mod_Flat (5 %)	Deep (216 cm)	0.0	4,346.1	0.0	123.9	12,524.8
Moderate (8 %)	Shallow (66 cm)	188.6	1,21,001.6	970.8	14,769.6	58,883.9
Moderate (8 %)	Mid (99 cm)	1.5	10,504.4	53.7	411.5	19,068.1
Moderate (8 %)	Deep (216 cm)	0.0	6,049.4	0.0	885.7	17,290.7

^a^The bracket in slope column means the average slope in each hillslope and

^b^That in soil column means the restrictive soil layer depth

^c^Agric_NT means agricultural land areas with no tillage

^d^Agric_CT means agricultural areas with conventional tillage

The LREW landuse map (Fig. 3) was created from Landsat Thematic Mapper data of 2003 with 30 × 30 m resolution. Subsequent GIS analyses were constrained by this resolution. As a result, each of the 20 subwatersheds in LREW was divided into 30 × 30 m grid cells. Each of the grid cells was assigned an annual erosion rate from Table 2 based on its unique combination of slopes, land use, and soils data. The sum of the grid cell erosion rates within a subwatershed was then aggregated as the subwatershed erosion rate (SLOAD_j_). The subwatersheds were then ranked with the greatest erosion rate receiving the highest rank. In order to validate this approach, we compared the HCT rankings to rankings developed from sediment load predictions by the soil and water assessment tool (SWAT, Arnold and others 1998) model (Cho and others 2010b). The SWAT study estimated annual average erosion rates from each subwatershed for a nine year simulation period (1996–2004).

**Fig. 5:**
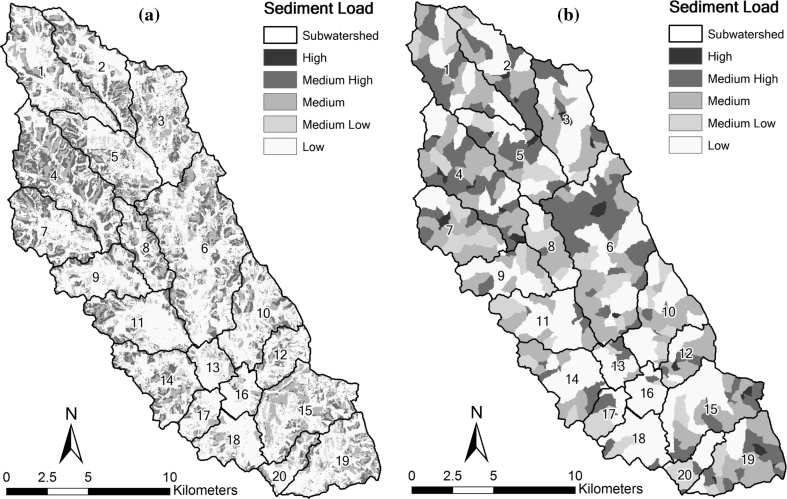
Comparison of the erosion rates resulting from **a** the HCT model based on the 30 × 30 m grid cells and **b** the SWAT model based on hydrologic response units (HRUs)

Figure 5 provides a visual comparison of the erosion rates resulting from the HCT and SWAT models. The HCT map displays the erosion rates for each of the 30 × 30 m grid cells while the SWAT map displays the erosion rates for hydrologic response units (HRUs). HRUs are areas (polygons) containing a unique combination of soil and land use and may vary greatly in size. For each model, the erosion rates from these dissimilar unit areas are aggregated to provide subwatershed erosion rates.

Table 3 presents the subwatershed rankings for total soil erosion (kg/year) and soil erosion per unit area (kg/ha-year) resulting from the HCT and SWAT models. Total soil erosion rankings show similar results for both models. In contrast, soil erosion per unit area rankings shows some discrepancies between the two models. These differences are likely caused by the unit scale difference between the two models discussed in the previous paragraph and by the limited combinations of slope, depth to restrictive layer, and land use utilized by the HCT simulations. Overall, the rankings are quite similar and indicate that the approach taken with the HCT model is acceptable for our prioritization model.

**Table 3:** Comparison of the subwatershed rankings for total amounts of soil erosion (kg/year) and soil erosion per unit area (kg/ha-year) resulting from the hydrologic characterization tool (HCT) and soil and water assessment tool (SWAT) models

Subwatershed number	Area (ha)	Rank by total soil erosion		Rank by soil erosion per area	
		HCT	SWAT	HCT	SWAT
1	2277	**3**	**3**	10	7
2	1770	**6**	7	**4**	**4**
3	2197	**5**	8	8	10
4	2791	**2**	**2**	**1**	**1**
5	1822	8	**6**	7	**5**
6	4609	**1**	**1**	**6**	**3**
7	1623	7	9	**3**	9
8	978	13	15	**2**	13
9	1333	14	14	16	15
10	1834	11	11	14	17
11	1722	10	12	11	18
12	848	15	13	15	**6**
13	582	20	19	20	16
14	1582	9	10	**5**	12
15	2222	**4**	**5**	9	8
16	584	18	20	17	20
17	572	19	18	18	14
18	1041	16	16	19	19
19	1703	12	**4**	12	**2**
20	565	17	17	13	11

The *bold letter* indicates the top 30 % of overall subwatershed ranks

### Marginal Change in Total Sediment Load per Conservation Dollar Invested, dSL/d$

**Fig. 6:**
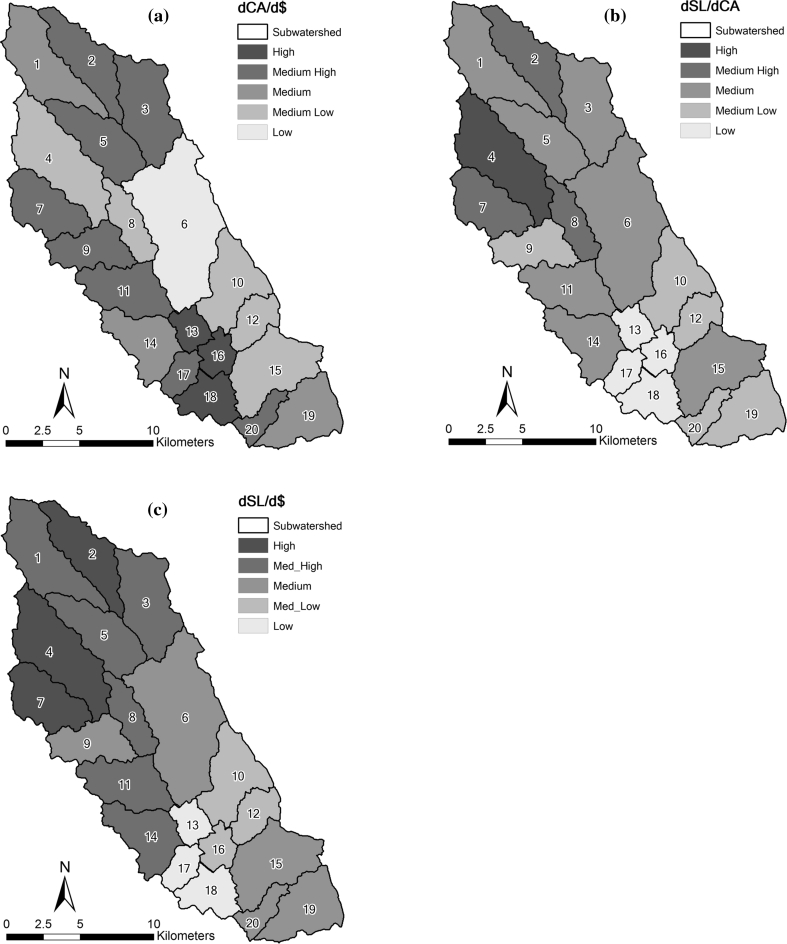
Comparison of mapped ranks for **a** the marginal change in conserved area per conservation dollar (dCA/d$), **b** the marginal change in sediment load per conserved area (dSL/dCA), and **c** the marginal change in total sediment load per conservation dollar (dSL/d$) in the LREW, which are based on uniform conservation cost indicator. The *numbers* on the map indicate subwatershed number, and rank is indicated by *color*. *High ranks* indicate high conservation priority (Color figure online)

Marginal change in total sediment load per conservation dollar invested was calculated using Eq. 1. The mapped ranks of dCA/d$, dSL/dCA, and dSL/d$ within each subwatershed of the LREW are shown on the left, middle, and right of Fig. 6, respectively. We classified the distribution of ranks by the Fisher-Jenks procedure for determining natural break classes (Jenks 1967). It is preferred to a quantile or equal area approach as it defines classes based on a distribution pattern (Schweiger and others 2002). The numbers on the map indicate subwatershed number—not rank. Rank is indicated by color. The three subwatersheds (2, 4, and 7) identified as having the highest potential marginal change in total sediment load per conservation dollar invested all have a relatively high proportion of cultivated land using conventional tillage (Fig. 3) and are, therefore, more susceptible to erosion. The proportion of agricultural land in subwatershed 2, 4, and 7 is 32.2, 53.9, and 39.1 %, respectively. The proportion of fallow land in these subwatersheds ranges from 1.4 to 3.4 %. These results, visualized as maps, can be used to screen and reduce the number of subwatersheds that need further assessment by decision-makers and managers at agencies such as NRCS.

The values of dCA/d$ in subwatersheds 4 and 7 are in the low to medium range because there is relatively little measurable conservation activity. In contrast, subwatershed 2 is ranked in the medium–high category because according to the data, a relatively large proportion of its area is protected by conservation easements. This indicates the community support and willingness for conservation activities to consider implementation of BMPs which prevent erosion. The high overall ranks of these three subwatersheds is primarily driven by the relatively high value of their dSL/dCA term compared with their CA/d$ term. Table 4 presents the calculated correlation between terms and land use. We used spreadsheet software to examine the correlation between the two sets of data. The correlation coefficients clearly show that the presence of agricultural land with conservation tillage was the most important parameter in this test application of the prioritization model. Likewise, the dSL/dCA term drove the ranking of the watersheds. In contrast, there was very low correlation between the dCA/d$ term and the final rankings. This is mostly because our test application was done on a small watershed within which we could not differentiate between some of the indicators in the dCA/d$ term.

**Table 4:** Comparison of the correlation coefficients between agricultural land with conservation tillage and each term

Correlation	dCA/d$	dSL/dCA	dSL/d$
Forest	0.55	0.44	0.29
Fallow	0.06	0.06	0.04
Grass	0.24	0.50	0.38
Agric_NT^a^	0.14	0.01	0.05
Agric_CT^b^	0.42	0.89	0.72
dCA/d$	–	–	0.09
dSL/dCA	–	–	0.90

^a^Agric_NT means agricultural land areas with no tillage and

^b^Agric_CT means agricultural areas with conventional tillage

## Discussion

This prioritization model was developed to provide agencies such as NRCS with a tool for identifying watersheds in which conservation practice implementation is likely to provide the most water quality improvement per conservation dollar invested. The model includes quantitative assessment of hydrologic processes as well as quantitative and qualitative assessment of socio-economic factors that may affect the prioritization process. Including implementation cost as an indicator helps to define the circumstances under which the results are applicable (McAllister and others 2000) and serves as an important constraining factor. In order to illustrate the importance of including implementation cost, we ran the model with the cost indicator fully implemented and with that indicator set at a uniform cost for all the subwatersheds. Table 5 represents the ranks of dCA/d$, dSL/dCA, and dSL/d$ under these two scenarios. The dSL/dCA term is the same for both scenarios. As described earlier, the local NRCS office provided information on BMP implementation costs within LREW for the two practices (terraces and grassed waterways) we included in this application. Table 5 shows that the rankings are quite different with the inclusion of the cost indicator. It is, therefore, quite important that reasonable estimates of implementation costs are obtained for individual watersheds and for terrain features. Indicator data should always be evaluated for accuracy and usefulness relative to the assessment objectives using clearly established protocols (Vellidis and others 2003a).

**Table 5:** The ranks comparison of dCA/d$, dSL/dCA, and dSL/d$ under two scenarios with the cost indicator fully implemented and with indicator set at a uniform cost for all the subwatersheds

Subwatershed number	Conservation cost indicator is fully implemented		Rank of dSL/dCA	Uniform conservation cost Indicator	
	Rank of dCA/d$	Rank of dSL/d$		Rank of dCA/d$	Rank of dSL/d$
1	17	16	10	12	9
2	11	12	**4**	**4**	**3**
3	16	15	8	10	7
4	20	18	**1**	15	**1**
5	**2**	**1**	7	8	**5**
6	**4**	**2**	**6**	20	14
7	12	11	**3**	7	**2**
8	13	10	**2**	17	**4**
9	8	7	16	**5**	13
10	18	20	14	19	17
11	10	8	11	11	8
12	9	13	15	18	15
13	**3**	**4**	20	**3**	20
14	14	14	**5**	14	**6**
15	19	19	9	16	10
16	**1**	**3**	17	**2**	16
17	**5**	**6**	18	**6**	19
18	**6**	9	19	**1**	18
19	15	17	12	13	12
20	7	**5**	13	9	11

The *bold letter* indicates the top 30 % of overall subwatershed ranks

The application of our conceptual model does not allow us to quantify sediment load reduction per dollar invested. Instead, our conceptual model is primarily a prioritization tool and can only produce a relative ranking of sediment reduction across watershed or subwatersheds. Use of the synoptic approach, based on this sediment load reduction model, is appropriate for prioritizing conservation efforts because it can minimize costs while maximizing information when funds are not available for more detailed assessments.

Data for a synoptic approach can come from multiple sources and are found in a variety of formats including tabular data, computerized databases, and mathematical predictive models (Abbruzzese and Leibowitz 1997; Vellidis and others 2003a). In addition, the best professional judgment is occasionally used in the absence of data. Consequently, the results of synoptic approaches are sometimes questioned. In order to reduce ambiguity in our model we selected only descriptors and indicators which are well supported in the literature (Norton and others 2009) and for which data are available. In addition, we followed the JSEM approach developed by Hyman and Leibowitz (2001) for evaluating indicators and developing the specific mathematical relationship between indicators.

Although our ranking results are an approximation of reality, the results cannot be treated as scientific findings. Prior to allocation of resources for BMP implementation, additional verification of the highest ranked watersheds must be done either through ground-truthing or the application of more sophisticated watershed transport models (Schweiger and others 2002).

The reliability of our model’s results could be enhanced by better populated and vetted region-wide datasets for our measurement endpoints. Defining the weighting factors associated with indicators such as WP, ENVG, and PREA through surveys of relevant professionals, managers, and other stakeholders would further reduce the uncertainty of results. Developing additional indices that address other human interventions or ecosystem functions would ensure more complete description for prioritizing conservation activities (McAllister and others 2000). However, these indices can only be included if datasets to support them become available.

CEAP is a multi-agency effort to quantify the environmental effects of conservation practices and programs and develop the science base for managing the agricultural landscape for environmental quality. CEAP findings will be used to guide USDA conservation policy and program development and help conservationists, farmers, and ranchers make more informed conservation decisions (Duriancik and others 2008; Maresch and others 2008; Osmond 2010). The prioritization model described here is one deliverable of this effort and may improve the efficacy of conservation practices and programs. In order to illustrate this potential, we are currently applying our prioritization model to the ecoregion of the southeastern Coastal Plain which contains the LREW and is shown in Fig. 2. Since our model performed well within the LREW, we can assume that it will also perform well within the ecoregion.

## Conclusions

The goal of this work was to develop a model for prioritizing watersheds within which agricultural BMPs can be implemented to reduce sediment load at the watershed outlets. The model considers both biophysical and socio-economic factors which affect the implementation of agricultural BMPs and ranks candidate watersheds within an ecoregion or river basin. The model is not a process-based simulation tool so the rankings only indicate which watersheds may provide the most cost-effective water quality response to the implementation of a suite of BMPs best-suited to control erosion. However, the application of the model to the LREW demonstrated that the model represents the physical drivers of erosion and sediment loading well. The model does not evaluate the water quality effect of the BMPs and it is incumbent on the model’s users to select the BMPs most suitable for the area under consideration.

The selected BMPs only affect the socio-economic component of the model through the cost of implementing the selected conservation practices. The model can be applied to many scales ranging from areas within a relatively small watershed to watersheds within an ecoregion or river basin. It is most effective when applied at the ecoregion or river basin scale. When applied to smaller watersheds, the tool is less effective at assessing the socio-economic factors that may drive the implementation of conservation practices because there may be little difference in these factors within a relatively small area.

The model was developed as a tool for prioritizing BMP implementation efforts in the watersheds of ecoregions associated with CEAP watersheds and will be first applied to the southeastern coastal plain ecoregion containing the LREW. This same approach we used in LREW can be used to build confidence in our model by policy makers at agencies such as NRCS. The model can be tested on the CEAP watersheds that have been intensively studied across the USA and then applied to the ecoregions that these watersheds represent. The goal of applying the tool to each ecoregion would then be to prioritize the watersheds within the ecoregion so that available conservation dollars can be used most effectively to improve water quality. A phosphorus load reduction version of the prioritization tool is currently under development.
